# Comprehensive analysis of a homeobox family gene signature in clear cell renal cell carcinoma with regard to prognosis and immune significance

**DOI:** 10.3389/fonc.2022.1008714

**Published:** 2022-10-31

**Authors:** Di Zheng, Jinzhuo Ning, Yuqi Xia, Yuan Ruan, Fan Cheng

**Affiliations:** Department of Urology, Renmin Hospital of Wuhan University, Wuhan, China

**Keywords:** homeobox family gene, signature, prognosis, immune microenvironment, ccRCC

## Abstract

The homeobox (HOX) family genes have been linked to multiple types of tumors, while their effect on malignant behaviors of clear cell renal cell carcinoma (ccRCC) and clinical significance remains largely unknown. Here, we comprehensively analyzed the expression profiles and prognostic value of HOX genes in ccRCC using datasets from The Cancer Genome Atlas (TCGA) and International Cancer Genome Consortium (ICGC) databases. We developed a prognostic signature comprising eight HOX genes (*HOXB1*, *HOXA7*, *HOXB5*, *HOXD8*, *HOXD9*, *HOXB9*, *HOXA9*, and *HOXA11*) for overall survival prediction in ccRCC and it allowed patients to be subdivided into high- and low-risk groups. Kaplan-Meier survival analysis in all the internal and external cohorts revealed significant difference in clinical outcome of patients in different risk groups, indicating the satisfactory predictive power of the signature. Additionally, we constructed a prognostic nomogram by integrating signature-derived risk score and clinical factors such as gender, age, T and M status, which might be helpful for clinical decision-making and designing tailored management schedules. Immunological analysis revealed that the regulatory T cells (Tregs) infiltrated differently between the two subgroups in both TCGA and ICGC cohorts. ssGSEA method showed that the enrichment scores for mast cells were significantly lower in high-risk group compared with the low-risk group, which was consistent in both TCGA and ICGC cohorts. As for the related immune function, the enrichment scores of APC co-inhibition, para-inflammation, and type II IFN response were consistently lower in high-risk group in both cohorts. Of the eight HOX genes, the mRNA and protein levels of HOXD8 were downregulated in ccRCC than that in normal tissues, and decreased expression of HOXD8 was associated with increased tumor grade and stage, and lymph node metastasis. Survival analysis revealed that lower expression of *HOXD8* predicted worse overall survival in ccRCC. In conclusion, our HOX gene-based signature was a favorable indicator to predict the prognosis of ccRCC cases and associated with immune cell infiltration. *HOXD8* might be a tumor suppressor gene in ccRCC and a potential predictor of tumor progression.

## Introduction

Renal cell carcinoma (RCC) is a common malignancy affecting urinary system, with a worldwide incidence rate growing 2% annual (1, 2). Clear cell renal cell carcinoma (ccRCC), characterized by robust lipid and glycogen accumulation, is the most frequent histological subtype of RCC, accounting for eighty to ninety percentage of all RCC cases. As one of the most lethal malignancies of the urological system, ccRCC is known for its high mortality rate and it causes around 175000 deaths per year worldwide (3). Early diagnosis and surgical resection could effectively improve clinical outcome for localized ccRCC, while approximately 30% of patients have developed metastasis when they are first diagnosed (4, 5). Besides, about 30%-35% ccRCC patients showed local recurrence or distant metastasis after nephrectomy (6). For relapsed or advanced RCC, patients typically undergo surgery and/or receive systemic therapy. Cytoreductive nephrectomy before systemic therapy is recommended in select patients with a potentially surgically resectable primary tumor mass (7). Patients with metastatic RCC who present with hematuria or other symptoms related to the primary tumor should be offered palliative nephrectomy if they are surgical candidates (7). Targeted therapy including tyrosine kinase inhibitors (TKIs; e.g., axitinib, cabozantinib, lenvatinib), and/or anti-VEGF antibodies are wildly used in first- and second-line treatments. The immune checkpoint inhibitors (ICIs; e.g., pembrolizumab, nivolumab) therapy, a method that can improve body’s anticancer immune response by regulating the activity of immune cells, provided a revolution in treatment options and have also been increasingly recommended and investigated (8). According to the NCCN guidelines for kidney cancer, combination of TKI with ICI, including axitinib with pembrolizumab, cabozantinib with nivolumab, and lenvatinib with pembrolizumab, were regarded as first-line preferred regimens for relapsed or advanced ccRCC (7). Nevertheless, due to the extensive heterogeneity in genomic level and the existence of a highly heterogeneous tumor microenvironment, prediction patients’ respond to these therapies remains a fundamental problem and patients’ prognosis varies even they share similar clinicopathological features and are under standard management. Exploring novel and reliable indicators to predict prognosis and response to therapies are of great importance for developing tailored management schedules and clinical decision-making, which may assist improving the prognosis of ccRCC patients.

The homeobox (HOX) genes encode a highly conserved family of transcription factors in mammal that are essential for organogenesis and development (9). Up to now, a total of thirty-nine HOX genes have been identified in human genome. On the basis of sequence similarity and chromosomal location, HOX genes are split into four clusters, namely HOXA, HOXB, HOXC, and HOXD, which are located on chromosomes 7, 17, 12, and 2, respectively (10). Over the past decades, we have come to discovered that many genes controlling embryogenesis such as HOX genes participate in carcinogenesis likewise (11). Apart from their role as master regulators of embryonic development in physiological status, HOX genes have been linked to multiple types of tumors (12–14). Altered expression of HOX genes were oncogenes or tumor suppressor genes by acting as transcription activator or transcriptional repressor, depending on context. In tumors, the deregulation of HOX genes may affect cell proliferation, invasion, differentiation, angiogenesis, and intracellular signal transduction (15–17). For example, higher HOXB9 expression was associated with poorer prognosis in adrenocortical carcinoma and simultaneous overexpression of HOXB9 and Ctnnb1 in adrenal cortex of transgenic mice led to larger adrenal tumors (18). In gastric cancer, the upregulated HOXA10 promoted the transcription of TGFB2, which triggered the activation of TGFβ/SMAD signaling and led to accelerated lung metastasis (19). In ccRCC, little is known about the role of HOX genes on malignant behaviors and its clinical significance.

The rapid development of high-throughput sequencing technology and bioinformatic methods has permitted their widespread application in cancer research, resulting in a comprehensive understanding of genetic or epigenetic abnormalities during carcinogenesis and progression (20, 21). Many of these abnormalities were confirmed to be potential therapeutic targets and prognosis indicators in multiple types of cancers in the later research. Recently, re-analyzing publicly available statistics such as RNA-Seq data from public databases has opened the door to the discovery of novel biomarker molecules, particularly certain gene families, for overall survival prediction in cancers (22, 23). In this study, using the transcriptome data of ccRCC sample and corresponding clinical information from public databases, we systematically analyzed the expression profiles and prognostic value of HOX genes in ccRCC. We developed an eight HOX gene-based signature for overall survival prediction and validated its accuracy in both internal and external cohorts. Additionally, we constructed a prognostic nomogram by integrating the signature-derived risk score and clinical parameters such as gender, age, T and M status for clinical decision-making. Moreover, we analyzed the association of the signature with immune microenvironment and distinct immune cell infiltration in ccRCC. Finally, we compared the expression of the eight HOX gene in tumor and adjacent normal tissues, and performed Kaplan-Meier survival analysis in ccRCC cohorts.

## Materials and methods

### Data sources

We downloaded transcriptome profiles (HTSeq-FPKM) of 539 ccRCC tumor tissues and 72 non-tumor tissues, and corresponding clinical information of ccRCC patients from the TCGA database (https://portal.gdc.cancer.gov/) and named as TCGA cohort. The ICGC cohort containing gene expression matrix files and clinical data was obtained from the ICGC database (https://dcc.icgc.org/projects) and was utilized for external validation. Patients without overall survival time or survival status were excluded in the subsequent analysis. Finally, a total of 621 ccRCC including 530 cases from TCGA cohort and 91 cases from ICGC cohort was collected in our study.

### Construction and validation of the HOX family gene-based signature

First, we randomly split the TCGA cohort (entire cohort) into a training cohort and a testing cohort at a ratio of roughly 1:1. To reduce overfitting, in the training cohort, differentially expressed HOX family genes were submitted to LASSO (least absolute shrinkage and selection operator) Cox regression analysis with the *glmnet* package in R. Following that, a multivariate Cox regression analysis was carried out, which resulted in the development of a HOX family gene-based signature in ccRCC. The risk score derived from the signature was calculated by a liner combination of gene expression level (Expi) and associated coefficients (Coefi), with the formula: $\text{risk score }=\sum_{i=1}^{n}\left(Coefi*Expi\right)$ . We computed the risk score of all the cases in training, testing, entire, and ICGC cohorts, and it allowed patients to be classified as high- or low-risk based on the median risk score value in training cohort. Finally, Kaplan-Meier survival analysis and time-dependent receiver operating characteristic (ROC) curves analysis were used to determine the signature’ predictive power in training, testing, entire, and ICGC cohorts.

### Construction of a prognostic nomogram

Integrating the signature-derived risk score and clinical factors such as gender, age, T and M status, a prognostic nomogram was built by using *rms* package in R. Calibration curves were plotted in TCGA and ICGC cohorts to evaluate whether the nomogram’s predicted overall survival of ccRCC patients was close to the actual clinical outcome.

### Functional annotation and gene set enrichment analysis

Using the *edgeR* package in R software, we first identified genes that were differently expressed across high- and low-risk groups, with the criterion of FDR<0.05 and |log2FC| >0.5. Subsequently, these differentially expressed genes (DEGs) were subjected to Gene ontology (GO) and Kyoto Encyclopedia of Genes and Genomes (KEGG) enrichment analyses using DAVID online tool (https://david.ncifcrf.gov/), and a *P*. value less than 0.05 was considered as significantly enriched. Gene set enrichment analysis was conducted using the GSEA software (version 4.0.2) to unearth the underlying signaling pathways associated with the signature based on the KEGG terms. *P*. value<0.05 and |NES| >1 was set as the screening criterion of the enrichment results, and the results were visualized using *ggplot2* package in R.

### Evaluation of immune cell infiltration and immune function

The CIBERSORT algorithm was used to calculate the proportion of infiltrated immune cells in ccRCC samples based on gene expression matrixes (24, 25), and the abundance of 22 infiltrated immune cell types were then compared between high- and low-risk groups. Using the *GSVA* package in R, single-sample gene set enrichment analysis (ssGSEA) was applied to determine the enrichment scores of immune cells and associated immunological activities, which were then compared across high- and low-risk groups.

### Tissue collection

A total of 20 frozen tissue samples including 10 ccRCC tissues and 10 adjacent normal tissues were collected in Renmin hospital of Wuhan university between August 2020 and June 2022. All the samples were harvest after resection and stored at -80°C. The experiment with patient tissue specimens was authorized by the Ethics Committee of Renmin Hospital of Wuhan University.

### RNA isolation and qRT-PCR

RNA isolation and quantitative real-time PCR (qRT-PCR) were performed as previously described (26). The primer sequences were list as follow: *GAPDH*, forward, 5’-CCATCTTCCAGGAGCGAGAT-3’ and reverse, 5’-TGAGTCCTTCCACGATACCA-3’; *HOXD8*, 5’-CACAAGCTCCTGGTAGACGA-3’ and reverse, 5’-GCTCTGTCTTCCTCCAGCTC-3’.

### Statistical analysis

R software (version 4.1.0) was employed to conduct all the statistical analyses and was utilized for visualization of the results. Kaplan-Meier method and the log-rank test was used to compare the difference in overall survival between risk groups. Differences of multiple variables between risk groups were assessed using Student’s *t*-test or Wilcoxon test. If not otherwise stated, *P*. value less than was deemed statistically significant.

## Results

### Characterization of homeobox family genes

**Figure 1:**
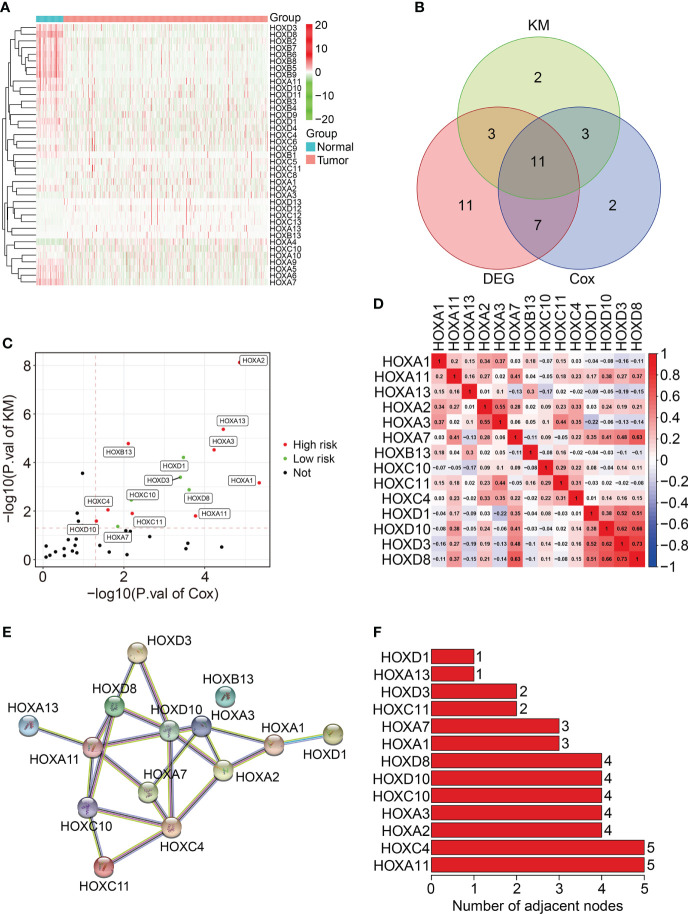
Characterization of homeobox family genes in ccRCC based on TCGA database. **(A)** Heatmap showing the expression patterns of HOX family genes in tumor tissues and adjacent normal tissues. **(B)** Venn plot showing the number of differentially expressed HOX genes and prognosis-related HOXs. **(C)** Volcano plot showing the prognosis-related HOXs based on univariate Cox regression analysis and Kaplan-Meier survival analysis. **(D)** Correlation heatmap of the 14 prognosis-related HOXs. **(E)** Protein-protein interaction network of the 14 prognosis-related HOXs. **(F)** Hub genes in the PPI network.

A total of thirty-nine homeobox family genes were enrolled in our study. The transcriptional expressions of these HOX genes in ccRCC tumor tissues and adjacent normal tissues were shown in **Figure 1A**. Of the 39 HOX family genes, thirty-two were differentially expressed between tumor and adjacent normal tissues (with the criteria of *P*-value less than 0.05) (**Figure 1B**). Moreover, fourteen HOX genes were significantly associated with the prognosis of ccRCC patients based on univariate Cox regression analysis and Kaplan-Meier survival analysis, and these genes were regarded as robust prognosis-related HOX genes (**Figures 1B, C**). Among the fourteen HOX genes, nine genes (*HOXA2*, *HOXA13*, *HOXA3*, *HOXB13*, *HOXA1*, *HOXA11*, *HOXC4*, *HOXC11*, and *HOXD10*) were risk factors (Hazard Ratio >1) and the other six genes (*HOXD1*, *HOXD3*, *HOXD8*, *HOXC10*, and *HOXA7*) were protective factors (Hazard Ratio<1) in ccRCC (**Figure 1C**). **Figure 1D** exhibits the correlation of these prognosis-related HOX genes. We then constructed a protein-protein interaction (PPI) network using the prognosis-related HOX genes (**Figure 1E**), and hub gene analysis suggested that *HOXA11* and *HOXC4* were the top two ranked genes in this PPI network (**Figure 1F**).

### Construction of a homeobox family gene-based signature in ccRCC

**Figure 2:**
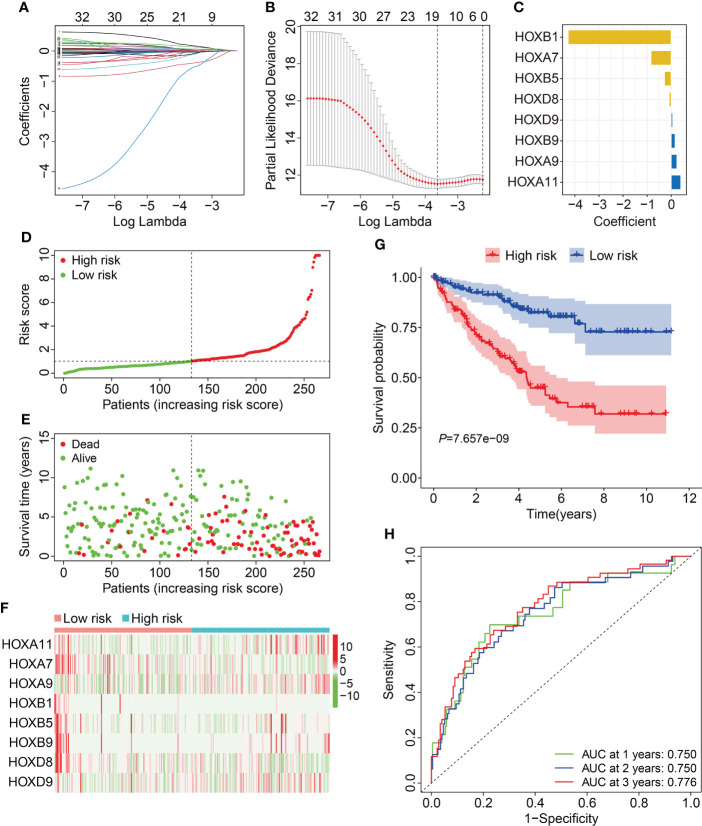
Construction of HOX family gene-based signature in ccRCC. **(A, B)** LASSO regression analysis and multivariate Cox analysis. **(C)** The distribution of the coefficient of the eight HOX family genes. **(D, E)** The distribution of risk score and survival status in high- and low-risk groups. **(F)** The transcription levels of the eight HOX family genes in high- and low-risk groups. **(G)** Kaplan-Meier survival curve for overall survival of patients in high- and low-risk groups. **(H)** Time-dependent ROC curve analysis in training cohort.

To construct a prognostic signature based on homeobox family genes, the TCGA ccRCC cohort was randomly classified into a training (*n*=266) and a testing cohort (*n*=264). In training cohort, the HOX family genes were subjected to LASSO regression analysis followed by multivariate Cox analysis (**Figures 2A–B**), and eight HOX genes (*HOXB1*, *HOXA7*, *HOXB5*, *HOXD8*, *HOXD9*, *HOXB9*, *HOXA9*, and *HOXA11*) were finally retained to construct a prognosis signature in ccRCC. The detailed information and coefficient of the eight HOX genes was shown in **Figure 2C** and **Table 1**. The risk score based on the prognosis signature was obtained by a linear combination of the expression levels of selected genes and corresponding coefficients. The formula was as follow: risk score = *HOXA11* × 0.401 + *HOXA7* × (-0.837) + *HOXA9* × 0.238 + *HOXB1* × (-4.284) + *HOXB5* × (-0.276) + *HOXB* × 0.163 + *HOXB9* × 0.163 + *HOXD8* × (-0.085) + *HOXD9* × 0.066. Then, the risk score of each patient in training cohort was computed and it allowed patients to be stratified into high- and low-risk groups according to the median value of risk score. **Figure 2D** shows the risk score distribution of patients in training cohort. The living status and survival time of patients in training cohort was exhibited in **Figure 2E**, and it suggested that the mortality rate of patients in high-risk group was higher than that in low-risk group. **Figure 2F** shows the transcription levels of the three HOX genes in high- and low-risk groups. Kaplan-Meier survival analysis demonstrated significant difference in the overall survival between high- and low-risk groups (**Figure 2G**). The area under the curve (AUC) values of the time-dependent receiver operating characteristic (ROC) curves were 0.750, 0.750, and 0.776 for 1-, 2- and 3-year overall survival, respectively (**Figure 2H**).

**Table 1:** Overall information of nine-HOXs constructing the prognostic model.

Gene Name	Coefficient	HR	HR.95L	HR.95H	P.value
HOXA11	0.4010	1.4933	1.1911	1.8721	0.0005
HOXA7	-0.8368	0.4331	0.2889	0.6493	0.0001
HOXA9	0.2382	1.2690	1.1587	1.3899	0.0000
HOXB1	-4.2839	0.0138	0.0000	4.4573	0.1462
HOXB5	-0.2765	0.7584	0.6211	0.9262	0.0067
HOXB9	0.1629	1.1769	1.0815	1.2807	0.0002
HOXD8	-0.0855	0.9181	0.8585	0.9818	0.0126
HOXD9	0.0662	1.0685	1.0336	1.1045	0.0001

### Validation of the homeobox family gene-based signature in internal cohorts

**Figure 3:**
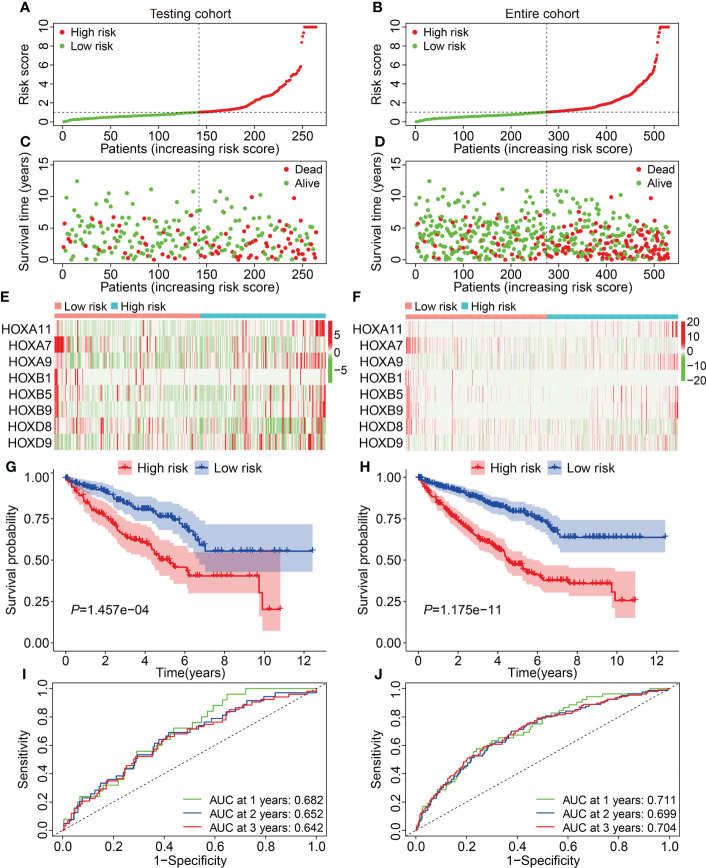
Validation of the HOX gene-based signature in internal cohorts. **(A, B)** The profile of risk score in testing cohort and entire cohort. **(C, D)** The distribution of survival time and status in testing cohort and entire cohort. **(E, F)** The expression patterns of the eight HOX genes in testing cohort and entire cohort. **(G, H)** Kaplan-Meier survival curve for overall survival of patients testing cohort and entire cohort. **(I, J)** Time-dependent ROC curve analysis in testing cohort and entire cohort.

**Figure 4:**
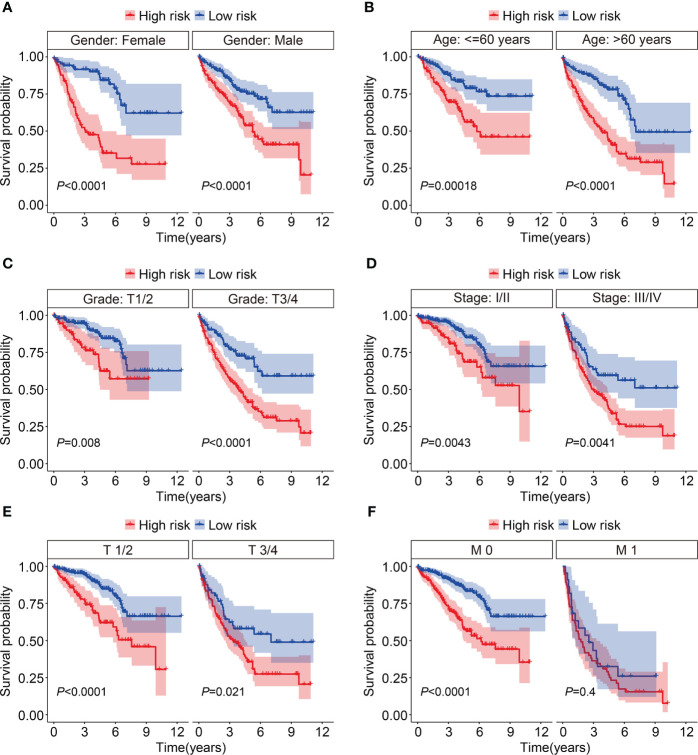
Kaplan-Meier survival curves to compare overall survival of high- and low-risk groups in subgroups stratified by gender **(A)**, age **(B)**, grade **(C)**, stage **(D)**, T and M status **(E, F)**.

First, we assessed the prognostic value of the HOX gene-based signature in internal cohorts including testing cohort and entire cohort. The risk score of each case in testing cohort and entire cohort was calculated using the formula mentioned above. Then, we divided patients of the internal cohorts into high- and low-risk groups using the median risk score value in training cohort as the cutoff. **Figures 3A, B** show the profile of risk score in testing cohort and entire cohort. The distributions of survival time and living status were shown in **Figures 3C, D**. The expression patterns of the three HOX genes were exhibited in **Figures 3E, F**. Kaplan-Meier survival analysis determined that patient in high-risk group had worse overall survival than that in low-risk group, which was consistent in both testing cohort and entire cohort (**Figures 3G, H**). Time-dependent ROC analyses suggested that the AUC values for 1-, 2-, and 3-year overall survival were 0.682, 0.652, and 0.642 in testing cohort (**Figure 3I**), and 0.711, 0.699, and 0.704 in entire cohort (**Figure 3J**), respectively. Moreover, we classified patients of the entire cohort into multiple subgroups according to the clinical parameters including gender (female *vs* male), age (≤60 *vs >*60), grade (Grade: T1/2 *vs* Grade: T3/4), stage (stage I/II *vs* stage III/IV), T (T 1/2 *vs* T3/4), and M stage (M0 *vs* M1). Survival analyses revealed that in different strata of clinicopathological features, patients of high-risk group harbored worse overall survival (**Figures 4A–F**), suggesting that our HOX family gene-based signature was quite useful and perform well in prognosis prediction.

### Validation of the homeobox family gene-based signature in external ICGC cohort

**Figure 5:**
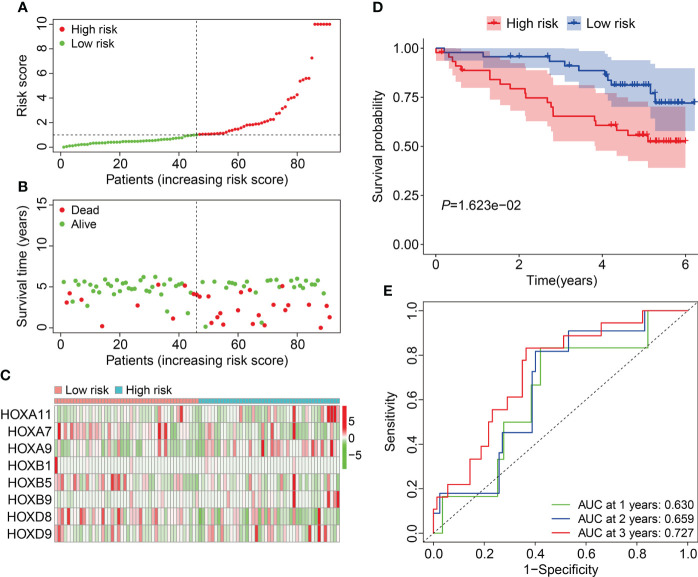
Validation of the HOX family gene-based signature in external ICGC cohort. **(A)** The profile of risk score in ICGC cohort. **(B)** The distribution of survival time and living status in ICGC cohort. **(C)** The expression patterns of the three HOX family genes in ICGC cohort. **(D)** Kaplan-Meier survival curve for overall survival of patients in ICGC cohort. **(E)** Time-dependent ROC curve analysis in ICGC cohort.

Subsequent, the external ICGC cohort was utilized to estimate the stability and generalizability of the prognostic signature. Using the same formula as in training cohort, the risk score of patients in ICGC cohort was computed and it allowed patients to be assigned into high- and low-risk groups based on the median value of risk score in training cohort. The risk score distribution of patients in high- and low-risk groups was shown in **Figure 5A**. The distribution of survival time and living status of patients in ICGC cohort was exhibited in **Figure 5B**, and it suggested that patients of high-risk group tended to have better survival status and longer survival time. **Figure 5C** shows the expression profile of the eight HOX genes in ICGC cohort. Survival analysis revealed that the overall survival of patients who belonged to the high-risk group was poorer than that of the low-risk group (**Figure 5D**). Time-dependent ROC analysis suggested that the AUC values were 0.630, 0.659, and 0.727 for 1-, 2-, and 3-year overall survival (**Figure 5E**). Taken together, these analyses indicated the satisfactory predictive power of the signature in forecasting the clinical outcomes of ccRCC patients.

### Estimation of the independent prognostic value of the signature and construction of a nomogram

**Figure 6:**
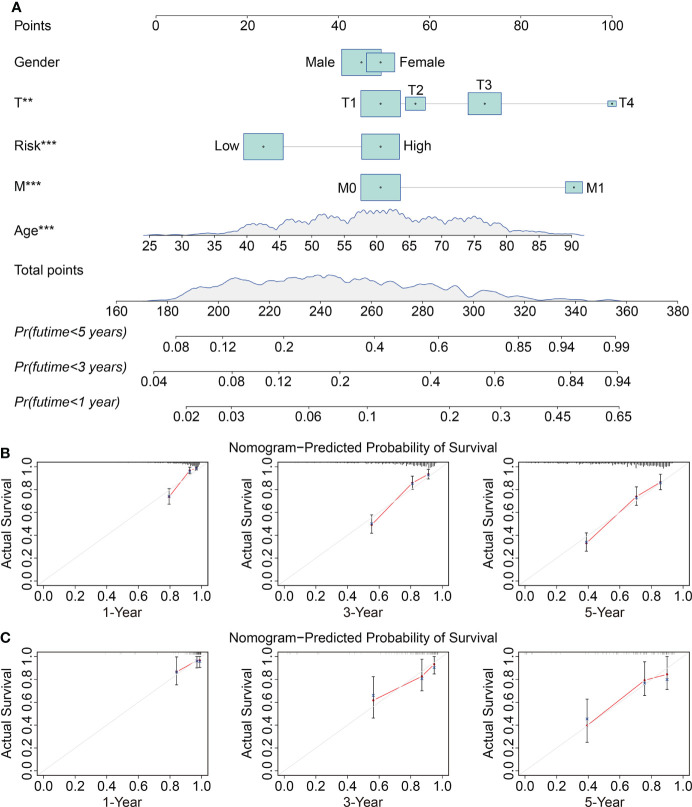
Construction and validation of a prognostic nomogram in ccRCC. **(A)** The nomogram combining risk score with clinical factors such as gender, age, T and M status for forecasting 1-, 3-, and 5-year overall survival. **(B, C)** The calibration plots of predicted and actual probabilities for the nomogram in TCGA and ICGC cohorts **P < 0.01; ***P < 0.001.

To investigate the independence of the signature and other clinicopathological parameters (age, gender, grade, stage, T and M status), both univariate and multivariate Cox regression analyses were performed. The results indicated that age, grade, stage, M status, and the signature-derived risk score showed significance in both analyses, and they thus could be regarded as independent prognostic indicators in patients with ccRCC (**Table 2**). Furthermore, a nomogram was created by combining risk score and other four clinicopathological characteristics including gender, age, T, and M status that were shared in TCGA and ICGC cohorts (**Figure 6A**). As shown in **Figures 6B, C**, calibration curves indicated satisfactory agreement between the nomogram prediction and actual observations, showing the remarkable dependability of the nomogram in predicting the overall survival of ccRCC patients.

**Table 2:** Univariable and multivariable analysis of the HOX family gene-based signature and clinical factors in the TCGA cohort.

Variables	Univariable analysis				Multivariable analysis				
	HR	95% CI of HR		*P*	HR	95% CI of HR		*P*
		Lower	Upper			Lower	Upper	
Age (≤60 vs >60)	1.788	1.309	2.441	0.000	1.694	1.233	2.329	0.001
Gender (Female vs Male)	0.930	0.679	1.274	0.651	0.932	0.673	1.290	0.671
Grade (I/II vs III/IV)	2.593	1.837	3.659	0.000	1.617	1.118	2.338	0.011
Stage (I/II vs III/IV)	3.610	2.618	4.978	0.000	2.158	1.039	4.481	0.039
T (T 1/2 vs T 3/4)	3.003	2.205	4.088	0.000	0.937	0.500	1.757	0.840
M (M0 vs M1)	4.205	3.070	5.759	0.000	2.447	1.655	3.616	0.000
Risk (High vs Low)	1.005	1.001	1.008	0.000	1.006	1.002	1.009	0.002

### Functional annotation of the HOX family gene-based signature

**Figure 7:**
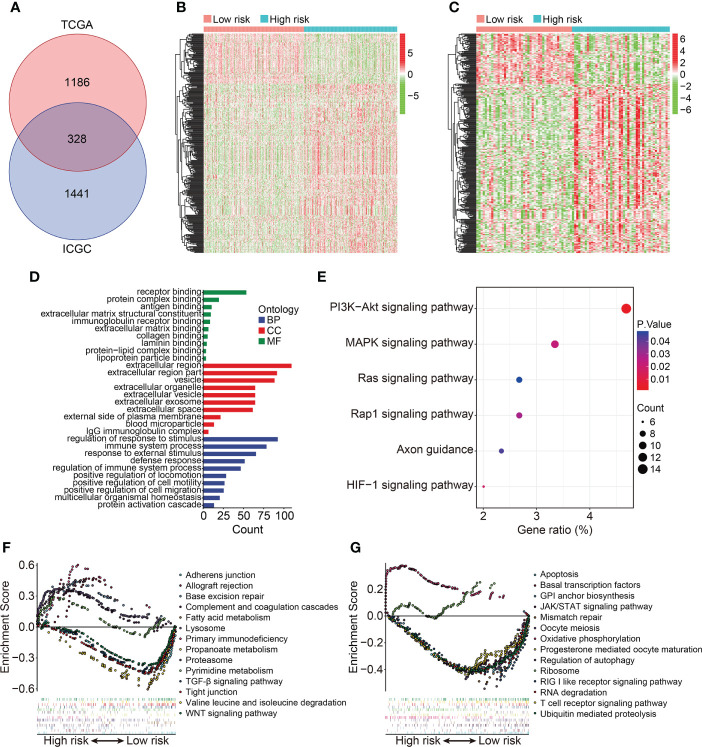
Identification of risk-related differentially expressed genes and functional enrichment analysis. **(A)** Venn plot exhibiting shared DEGs between different risk groups in TCGA and ICGC cohorts. **(B, C)** Heatmap showing the expression profiles of the DEGs in TCGA and ICGC cohorts. **(D, E)** GO and KEGG enrichment analyses. **(F, G)** Gene set enrichment analysis in TCGA and ICGC cohorts.

To reveal the underlying biological mechanism of the HOX family gene-based signature, we screened differentially expressed genes (DEGs) between high- and low-risk groups using *edgeR* filtration. A total of 328 shared DEGs between different risk groups in both TCGA and ICGC cohorts were identified the criterion of FDR<0.05 and |log2FC| >0.5 (**Figure 7A**). The expression patterns of these shared DEGs in TCGA and ICGC cohorts were exhibited in **Figures 7B, C**. Then, we annotated the function of these shared DEGs using DAVID database. GO enrichment analysis suggested that biological processes including regulation of response to stimulus, immune system process, response to external stimulus, defense response, and regulation of immune system process, were significantly enriched. As for the cellular component, extracellular region, extracellular region part, and vesicle were the three most enriched terms. In the molecular function category, DEGs were mainly enriched in receptor binding, protein complex binding, and antigen binding (**Figure 7D**). KEGG enrichment analysis suggested that multiple signaling pathways including PI3K-Akt, MAPK, Ras, Rap1, and HIF-1 were significantly enriched (**Figure 7E**). GSEA method revealed that allograft rejection, base excision repair, complement and coagulation cascades, lysosome, primary immunodeficiency, proteasome, and pyrimidine metabolism were markedly enriched in ccRCC samples with higher risk scores in TCGA cohort. Meanwhile, hallmarks including adherens junction, fatty acid metabolism, propanoate metabolism, TGF-β signaling pathway, tight junction, valine leucine and isoleucine degradation, and WNT signaling pathway were significantly enriched in ccRCC samples of low-risk group in TCGA cohort (**Figure 7F**). In ICGC cohort, oxidative phosphorylation and ribosome were significantly enriched in ccRCC samples of high-risk group, while hallmarks such as apoptosis, basal transcription factors, JAK/STAT signaling pathway, RIG I like receptor signaling pathway, and T cell receptor signaling pathway were markedly enriched in ccRCC samples of low-risk group (**Figure 7G**).

### Association between the HOX family gene-based signature with immune cell infiltration

**Figure 8:**
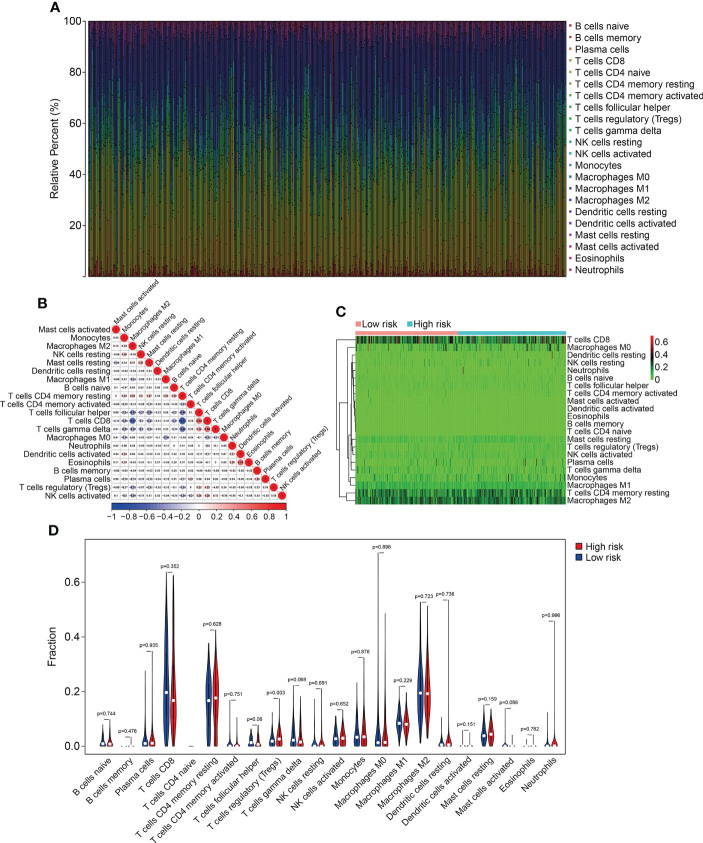
Comparison of immune cell infiltration in high- and low-risk groups in TCGA cohort. **(A)** Relative abundance of immunocyte infiltration in KIRC samples of the TCGA cohort. **(B)** The heatmap showing the correlation of infiltrating immune cells in the TCGA cohort. **(C, D)** The fraction of 22 immune cell types in high- and low- risk groups of the TCGA cohort.

**Figure 9:**
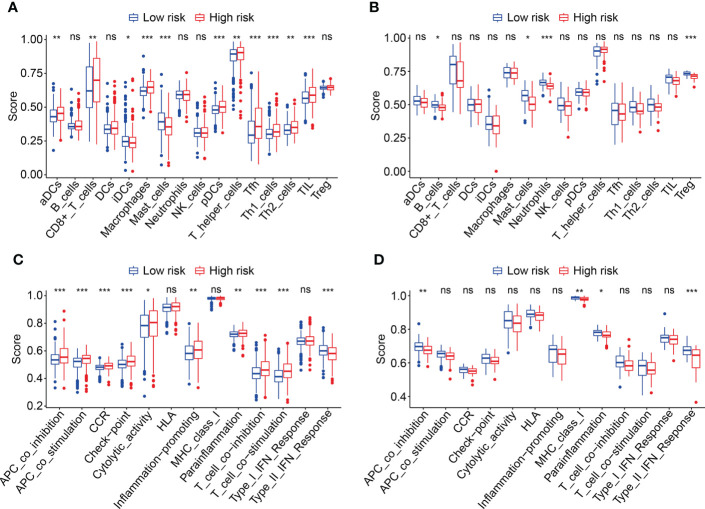
Comparison of immune cell infiltration and immune function based on ssGSEA. **(A, B)** Box plots exhibiting enrichment scores of immunocytes between the two subgroups in TCGA and ICGC cohorts. **(C, D)** Box plots exhibiting enrichment scores of the related-immune function between the two subgroups in TCGA and ICGC cohorts *P < 0.05; **P < 0.01; ***P < 0.001. not significant.

**Figure 10:**
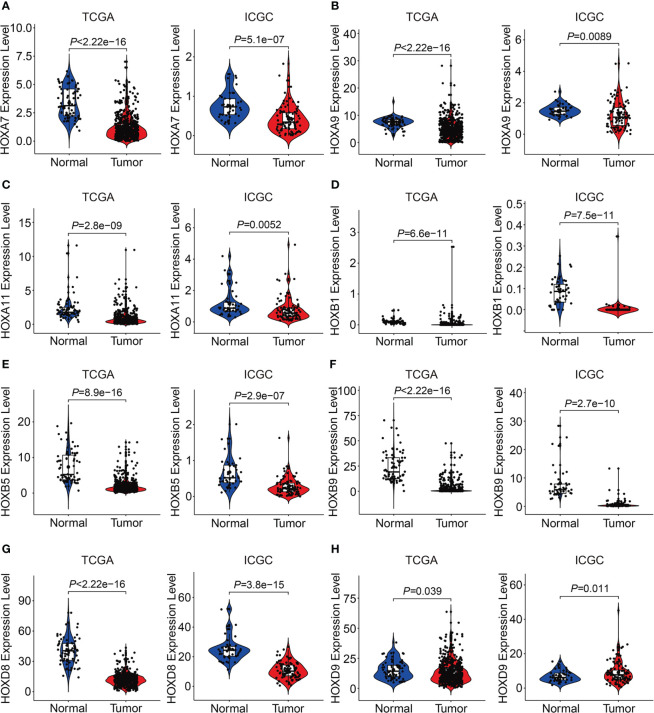
Comprising the expression of *HOXA7***(A)**, *HOXA9***(B)**, *HOXA11* **(C)**, *HOXB1***(D)**, *HOXB5***(E)**, *HOXB9* **(F)**, *HOXD8***(G)**, and *HOXD9* **(H)** between tumor tissues and adjacent normal tissues in TCGA and ICGC cohorts.

To explore the relationship between HOX family gene-based signature with the immune landscape of ccRCC, we estimated the proportions of immune cell infiltrated in each ccRCC sample by analyzing RNA sequencing data, and compared them between high- and low-risk groups. **Figure 8A** and **Supplementary Figure 1A** show the proportion of 22 infiltrated immune cell types in ccRCC samples of TCGA and ICGC cohorts, and it suggested that M2 macrophages, CD8 T cells, and resting memory CD4 T cells were the three most abundant immune cells in tumor microenvironment. The correlations of these infiltrated immune cells in ccRCC samples of TCGA and ICGC cohorts were shown in **Figure 8B** and **Supplementary Figure 1B**. In TCGA cohort, the regulatory T cells (Tregs) infiltrated differently between the two subgroups (**Figures 10C, D**). In ICGC cohort, a higher level of immune infiltration by regulatory T cells (Tregs), and a lower level of M1 macrophages and resting dendritic cell were associated with higher risk score (**Supplementary Figure 1C, D**). Additionally, we employed ssGSEA method to compare the enrichment scores of immune cell and related immune functions in high- and low-risk groups. As shown in **Figures 9A, B**, the scores for mast cells were significantly lower in high-risk group compared with the low-risk group, which was consistent in both TCGA and ICGC cohort. As for the related immune function, the enrichment scores of APC co-inhibition, para-inflammation, and type II IFN response were consistently lower in high-risk group in both cohorts (**Figures 9C, D**).

### Expression and Kaplan-Meier survival analysis of the eight HOX family genes

**Figure 11:**
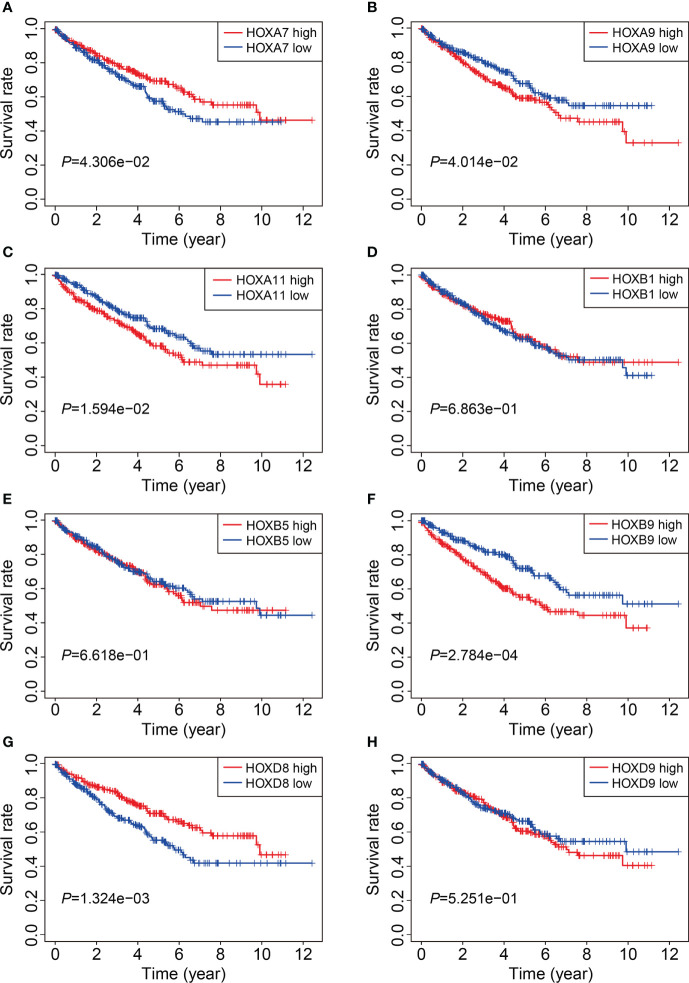
Kaplan-Meier survival analysis of *HOXA7***(A)**, *HOXA9***(B)**, *HOXA11* **(C)**, *HOXB1***(D)**, *HOXB5***(E)**, *HOXB9***(F)**, *HOXD8***(G)**, and *HOXD9* **(H)** in TCGA cohort.

We then analyzed the expression levels of the eight HOX family genes in ccRCC tissues and adjacent normal tissues, and performed Kaplan-Meier survival analysis in TCGA and ICGC cohorts. As shown in **Figures 10A-G**, the transcript levels of *HOXB1*, *HOXA7*, *HOXB5*, *HOXD8*, *HOXB9*, *HOXA9*, and *HOXA11* were significantly lower in ccRCC tumor tissues compared to adjacent normal tissues, which was consistent in both TCGA and ICGC cohorts. Compared to normal tissues, the expression of *HOXD9* was lower in ccRCC tumor tissues of TCGA cohort, while it was higher in ccRCC tumor tissues of ICGC cohort (**Figure 10H**). Meanwhile, Kaplan-Meier survival analysis in TCGA cohorts revealed that lower expression of *HOXA7* and *HOXD8*, and higher expression of *HOXA9*, *HOXA11*, and *HOXB9* were associated with worse overall survival in ccRCC patients (**Figures 11A–H**). In ICGC cohort, survival analysis indicated that higher expression of *HOXA9* predicted poorer prognosis in ccRCC (**Supplementary Figure 2A–H**).

### HOXD8 was downregulated in ccRCC and correlated with tumor progression

**Figure 12:**
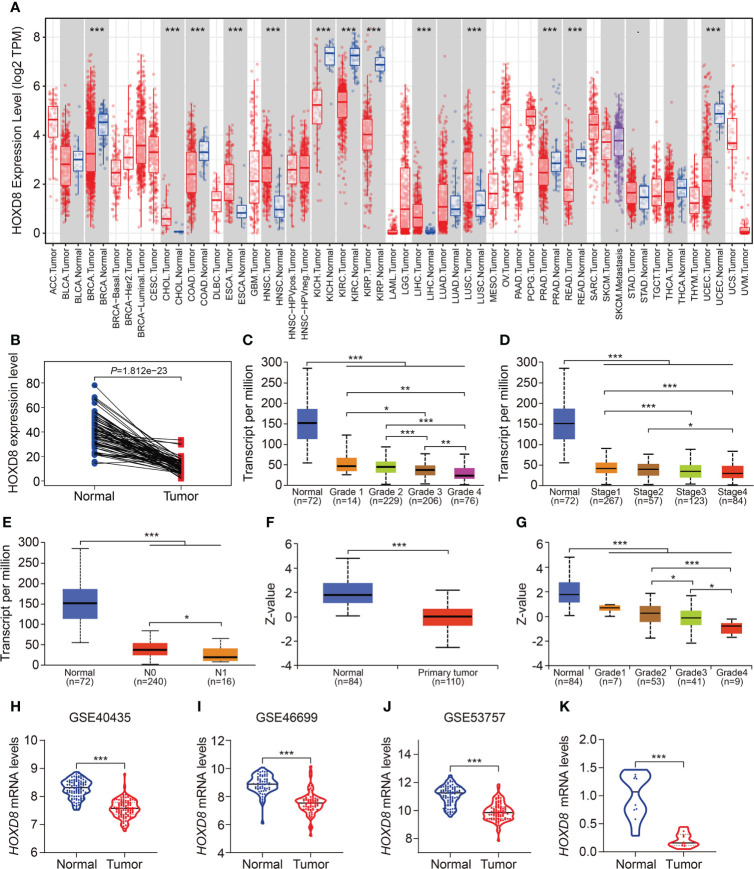
HOXD8 was downregulated in KIRC and correlated with tumor progression. **(A)** The expression profiles of HOXD8 in various types of tumors. **(B)** Comparison of the expression of HOXD8 in KIRC tissues and match non-tumor tissues. **(C-E)** The expression of HOXD8 in KIRC tissues with different tumor grade, stage, and N status. **(F)** The protein level of HOXD8 in KIRC tissues and normal tissues. **(G)** The protein level of HOXD8 in KIRC tissues with different tumor grade. **(H-J)** Comparison of the expression of HOXD8 in normal and tumor tissues in GSE40435, GSE46699, and GSE53757 database. **(K)** qRT-PCR was used to detect HOXD8 expression in clinical samples *P < 0.05; **P < 0.01; ***P < 0.001.

Finally, we comprehensively analyzed HOXD8 in ccRCC based on public resources. **Figure 12A** shows the expression profiles of *HOXD8* in various tumor types and it suggested that compared to adjacent normal tissues, *HOXD8* was downregulated in tumor tissues including BRCA, COAD, KIRC, KIRP, KICH, PRAD, READ, and UCEC, while it was upregulated in tumor tissues such CHOL, ESCA, HNSC, LIHC, and LUSC. Moreover, *HOXD8* expression were markedly downregulated in ccRCC tissues than that in match non-tumor tissues (**Figure 12B**). Besides, *HOXD8* expression was significantly decreased with the increase of tumor grade and stage, and lymph node metastasis (**Figures 12C–E**). Additionally, the protein level of HOXD8 was also lower in ccRCC tissues than that in normal tissues (**Figure 12F**), and HOXD8 protein level decreased with the increase of tumor grade (**Figure 12G**). Finally, we analyzed the expression of HOXD8 in three independent datasets (GSE40435, GSE46699, and GSE53757) from GEO database and performed qRT-PCR to detect *HOXD8* expression in clinical samples. Our results indicated that *HOXD8* expression were dramatically downregulated in ccRCC tissues compared to adjacent non-tumor tissues (**Figures 12H–K**).

## Discussion

Members of HOX family genes had been found to be aberrantly expressed in multiple types of tumors. In ccRCC, although some studies have indicated that dysregulation of HOX genes such as *HOXD1*, *HOXA13*, and *HOXC11* were associated with cell proliferation, metastasis, and apoptosis (27–29), while the detailed roles of HOX family genes on malignant behaviors of ccRCC and its prognostic values remained largely to be characterized. Here, we comprehensively analyzed the expression profiles and clinical significance of HOX genes in ccRCC using transcriptome profiles of tumor samples and corresponding clinical information from the TCGA database. We are suppressed to find that over eighty percent (32/39) of HOX genes were differentially expressed between ccRCC samples and adjacent normal tissues, and about thirty-five percent (14/39) of HOX genes were robustly associated with patients’ prognosis. These analyses indicated that HOX genes might exert vital role in the development and progression of ccRCC. Subsequently, we built a prognostic signature based on eight HOX genes including *HOXB1*, *HOXA7*, *HOXB5*, *HOXD8*, *HOXD9*, *HOXB9*, *HOXA9*, and *HOXA11* in ccRCC for risk stratification, which allowed patients with higher or lower risk score to be divided into different risk groups. Comparing the overall survival in subgroups of all the internal cohorts (training cohort, testing cohort, entire cohort) and external cohort (ICGC cohort) by Kaplan-Meier survival method indicated that the overall survival of patients who belonged to the high-risk group was poorer than that of the low-risk group. Moreover, time-dependent ROC curve analyses suggested the favorable forecasting performance of the signature. Besides, the specificity and accuracy of our eight-gene based signature was superior to some previously reported prognostic signatures in ccRCC (30, 31), in terms of AUC values of the ROC curves (**Supplementary Table 1**). Taken together, our HOX gene-based signature harbored satisfactory accuracy and generalizability in prognosis prediction. Additionally, univariate and multivariate Cox regression analyses revealed that the signature-derived risk score was an independent prognostic indicator in patients with ccRCC. Furthermore, we successfully developed a nomogram by combining signature-derived risk score, gender, age, T and M status to expand the predictive ability of the signature, which exhibited good clinical application value and might be helpful in facilitating individualized treatment and clinical decision-making.

In order to reveal the underlying biological mechanism of the HOX family gene-based signature, a total of 328 shared DEGs between the two risk groups were identified and were then functionally annotated. In KEGG enrichment analysis, we found that these DEGs were mainly enriched in PI3K-Akt, MAPK, Ras, Rap1, and HIF-1 signaling pathways, and these enriched pathways had been previously demonstrated to be critical for ccRCC development and progression (32–35). For example, the modestly mutated genes in PI3K/AKT pathway leads to its highly activated in ccRCC and represents promising drug targets (36). Isoform-specific AKT inhibitors are being tested in ccRCC clinical trials (37). Thus, we could speculate that the two risk groups stratified by our signature might exhibit distinct activation of these signaling pathways.

Tumor microenvironment consists of two major categories of components, including cellular components (e.g., tumor cell, vascular endothelial cells, immune cells, and mesenchymal stem cells) and surrounding acellular components (e.g., cytokines, adhesion molecules, growth factors). These non-tumor components provide a scaffold, barrier and environment for tumor occurrence and growth. Recent studies revealed that ccRCC is one of the most immune and vascularly infiltrated cancer types and the immune microenvironment played crucial role in ccRCC progression, and was associated with immune therapy response and patients’ prognosis (38, 39). Thus, we further explored the association of the signature with immune microenvironment and immune cell infiltration in ccRCC. CIBERSORT algorithm revealed that CD8 T cells, M2 macrophages, and resting memory CD4 T cells were the three most abundant immune cell types in ccRCC tissues. Moreover, a higher level of immune infiltration by regulatory T cells (Tregs), and a lower level of M1 macrophages and resting dendritic cell were associated with higher risk score. The regulatory T cells in tumor microenvironment hindered protective immunosurveillance of tumor and suppress anticancer immunity, thereby leading to tumor progression (40–42). A higher proportion of infiltrated regulatory T cells in tumor tissues was regarded to be associated with worse prognosis (43). Treg-cell targeting therapy was shown to evoke and enhance anti-tumor immune response (44). The M1 macrophages, developed from M0 macrophages, exert tumor inhibiting role by mediating cytotoxicity and antibody-dependent cell-mediated cytotoxicity (ADCC) to kill tumor cells (45, 46). The abundance of infiltrating M1 macrophages was positively correlated with clinical outcome in diverse tumor types (47). By combining our findings with those of previous studies, we were able to conclude that our HOX gene-based signature was closely associated with distinct immune status and different patterns of infiltrating immune cells, which might contribute to diverse clinical outcome in the two risk groups. Our signature might offer prominent therapy guidance and could be useful in determining which patients would benefit from immune therapy.

Of the eight HOX genes (*HOXB1*, *HOXA7*, *HOXB5*, *HOXD8*, *HOXD9*, *HOXB9*, *HOXA9*, and *HOXA11*) comprised in our signature, their transcript levels were consistently lower in ccRCC tissues compared to adjacent normal tissues (except for *HOXD9*). Survival analysis indicated that lower expression of *HOXA9*, *HOXA11*, and *HOXB9* were associated with favorable clinical outcome in ccRCC patients, thus the prognostic prediction performance of *HOXA9*, *HOXA11*, and *HOXB9* might be controversial with their expression level in ccRCC. *HOXA9* had been extensively studied in various types of tumors and it could act in opposite ways when it was dysregulated in tumors. Lower expression of *HOXA9*, accompanied by hypermethylation of its promoter region, was diagnostic or prognostic biomarker in tumors such as non-small cell lung cancer, ovarian cancer, and head and neck squamous cell carcinoma (48–50). Modulating HOXA9 expression could either promote or inhibit tumor progression through different mechanism, depending on context (51, 52). In renal cell tumors (RCT), promoter methylation of HOX9A was disclosed in 73% of RCTs, and the two-gene (HOX9A and OXR1) methylation panel led to 90% sensitivity and 98% specificity in the identification of ccRCC (53). However, up to now, little is known about the role of HOXA9 in ccRCC, further experiments should be carried out to detect the effect of HOXA9 knockdown or overexpression on malignant behaviors of ccRCC cells and unearth the underlying mechanism. *HOXA11* was a putative tumor suppressor in a number of solid tumors and it was frequently epigenetic inactivated (54, 55). HOXA11 antisense LncRNA (HOXA11-AS) was shown to be associated with advanced tumor stage and metastasis in RCC. Functionally, overexpression of HOXA11-AS promoted tumor growth and invasion through regulating miR-146b-5p-MMP16 axis (56). *HOXB9* was also reported to play a dual role in different types of tumors (57). The aberrant expression of *HOXB9* in tumors was not only prognostic predictor but also indicator of response to target therapy. Protein encoded by *HOXB9* functioned as oncoprotein and could accelerate cell proliferation and invasion in endometrial cancer, colorectal cancer, and hepatocellular carcinoma cells (58–60). However, HOXB9 could also delay tumor progression in other kinds of tumors such as gastric cancer and pancreatic cancer (61, 62). Nevertheless, the functional role of HOXB9 in ccRCC remains largely unknown and deserves further investigation. *HOXB1* is a well-defined tumor suppressor gene in diverse tumors (63, 64) and it was dramatically downregulated in ccRCC. However, *HOXB1* expression is extremely low in ccRCC tissues, which might limit its biological roles in ccRCC. Whether *HOXB1* had an effect on malignant behavior of ccRCC cells should be further explored *in vitro* and *in vivo*. The downregulated expression of *HOXA7* in ccRCC and its lower expression being associated with poorer patients’ prognosis indicated that it might be a tumor suppressor in ccRCC. However, *HOXA7* was recently more reported to be oncogene and promoted oncogenic characteristics in many kinds of tumors such as liver cancer, cervical cancer, ovarian cancer, colorectal cancer and breast cancer (65–69). The role of *HOXA7* in ccRCC had not been reported until now and exploring its effect on malignant characteristics of ccRCC might lead to the understanding of its diverse biological role and the complicated intracellular regulatory network. *HOXB5* and *HOXD9* were suspected to be oncogenes in tumors and their translation products were reported to aggravate malignant development of tumors (70–72). Though our bioinformatic analysis suggested that *HOXB5* and *HOXD9* were markedly downregulated in ccRCC, the detailed role of them in ccRCC should be further experimentally investigated. Protein encoded by *HOXD8* gene is a conserved transcription factor that exert a tumor-suppressing role in various tumors through diverse mechanism. Overexpression of HOXD8 in colorectal cancer cells impaired cell proliferation and migration *via* inducing apoptotic event (73). Enforced expression of HOXD8 in breast cancer repressed tumor growth by inactivating AKT/mTOR pathway (74). Up to now, the role of HOXD8 in ccRCC had not been elucidated. Intriguingly, we found that the mRNA and protein levels of HOXD8 were downregulated in ccRCC than that in normal tissues, and decreased expression of HOXD8 was associated with increased tumor grade and stage, and lymph node metastasis. Survival analysis revealed that lower expression of *HOXD8* predicted worse overall survival in ccRCC. Taken together, it is reasonable to speculate that HOXD8 might be a tumor suppressor gene in ccRCC and a potential predictor of tumor progression.

Inevitably, there are several shortcomings in our study. First, we should endeavor to collect prospective cohort to verify the reliability of our signature. Second, we need to examine the protein levels of the HOX family genes, especially *HOXD8*, in ccRCC though immunoblotting or immunohistochemistry staining. Third, the role of HOX family genes, especially *HOXD8*, are warrant to be experimentally explored in ccRCC.

In all, we here systemically analyzed HOX family genes in ccRCC using bioinformatic method, and successfully constructed a prognostic signature based on eight HOX genes. Our signature was a favorable indicator to predict the prognosis of ccRCC cases and associated with tumor immune microenvironment and immune cell infiltration. *HOXD8*, one of the eight HOX genes, might be a tumor suppressor gene in ccRCC and a potential predictor of tumor progression.

## Data availability statement

The original contributions presented in the study are included in the article/**Supplementary Material**. Further inquiries can be directed to the corresponding author/s.

## Ethics statement

The studies involving human participants were reviewed and approved by Ethics Committee of Renmin Hospital of Wuhan University. The patients/participants provided their written informed consent to participate in this study.

## Author contributions

FC and YR designed the study. DZ and JN conducted bioinformatic analysis, wrote the manuscript and responsible for language revisions. All authors contributed to the article and approved the submitted version.

## Funding

This study was funded by grants from National Natural Science Foundation of China (81870471 and 81800617) and Science and Technology Major Project of Hubei Province(2019AEA170).

## Acknowledgments

We sincerely thank the TCGA project for using their data.

## Conflict of interest

The authors declare that the research was conducted in the absence of any commercial or financial relationships that could be construed as a potential conflict of interest.

## Publisher’s note

All claims expressed in this article are solely those of the authors and do not necessarily represent those of their affiliated organizations, or those of the publisher, the editors and the reviewers. Any product that may be evaluated in this article, or claim that may be made by its manufacturer, is not guaranteed or endorsed by the publisher.
