# Supplementary material for: Comprehensive analysis of a homeobox family gene signature in clear cell renal cell carcinoma with regard to prognosis and immune significance
Source: Front Oncol. 2022 Oct 31;12:1008714. doi: 10.3389/fonc.2022.1008714 (PMC9660242; doi:10.3389/fonc.2022.1008714)
Supplement: Supplementary file 1 [file DataSheet_1.pdf]

Supplementary Material

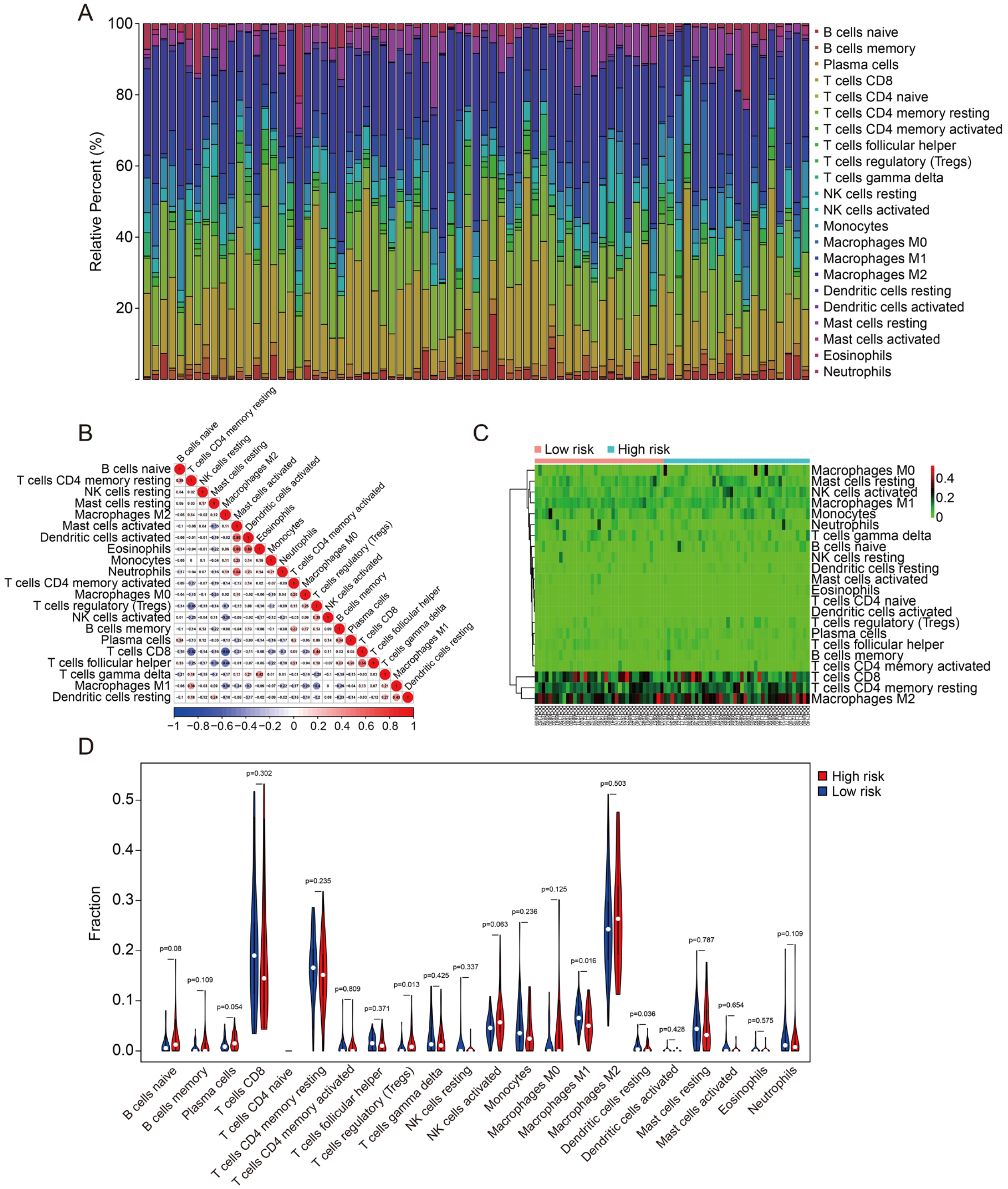

Supplementary Figure 1. Comparison of immune cell infiltration in high- and low-risk groups in ICGC cohort. (A) Relative abundance of immunocyte infiltration in KIRC samples of the ICGC cohort. (B) The heatmap showing the correlation of infiltrating immune cells in the ICGC cohort. (C-D) The fraction of 22 immune cell types in high- and low- risk groups of the ICGC cohort.

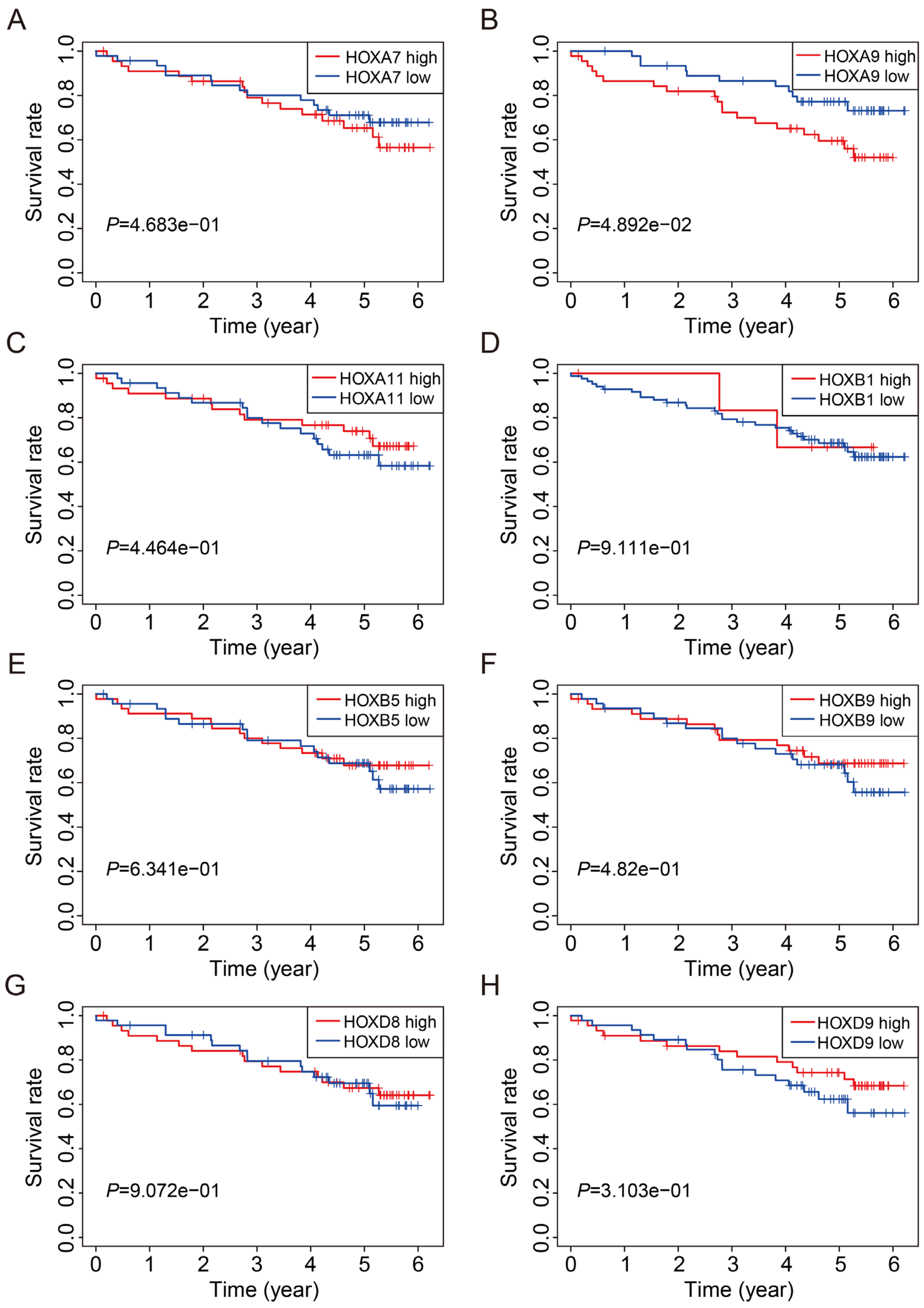

Supplementary Figure 2. Kaplan-Meier survival analysis of *HOXA7* (A), *HOXA9* (B), *HOXA11* (C), *HOXB1* (D), *HOXB5* (E), *HOXB9* (F), *HOXD8* (G), and *HOXD9* (H) in ICGC cohort.

Supplementary Table 1. Comparisons between the eight-gene signature and previous studies

Author	Published online data	PMID	Gene signature composition	AUC values		
				1-year	2-year	3-year
HOX-signature	--	--	*HOXB1, HOXA7, HOXB5, HOXD8, HOXD9, HOXB9, HOXA9, HOXA11*	0.750	0.750	0.776
Chen *et al*	2022 Jan	35165523	*FASLG*, *TLR3*, *ZBP1*	0.707	NA	0.635
Zhan *et al*	2021 Apr	33968978	*AURKB*, *FOXM1*, *PTTG1*, *TOP2A*, *TACC3*, *CCNA2*, *MELK*	0.695	0.687	0.678
Zhou *et al*	2022 Aug	35808868	*LINC00460*, *LINC01094*, *AC008870.2*, *AC068792.1*, *AC007637.1*	0.738	NA	0.668
Xing *et al*	2022 May	35619698	*CCR4*, *CMTM3*, *IFITM1*, *MX2*, *NR3C2*	0.736	NA	0.746
Ning *et al*	2022 Jan	35141153	*GPC3, KIF5A, PLAUR, ANKZF1, ETS1, SELENBP1.*	0.711	NA	0.708

NA: missing or unknown values.
